# Non-Hodgkin's lymphoma, obesity and energy homeostasis polymorphisms

**DOI:** 10.1038/sj.bjc.6602762

**Published:** 2005-09-13

**Authors:** E V Willett, C F Skibola, P Adamson, D R Skibola, G J Morgan, M T Smith, E Roman

**Affiliations:** 1 Epidemiology and Genetics Unit, Department of Health Sciences, Seebohm Rowntree Building, University of York, York YO10 5DD, UK; 2 Division of Environmental Health Sciences, School of Public Health, 140 Earl Warren Hall, University of California, Berkeley, CA 94720-7360, USA; 3 Institute of Cancer Research, The Royal Marsden, Downs Road, Sutton, Surrey SM2 5PT, UK

**Keywords:** non-Hodgkin's lymphoma, body mass index, SNP, leptin, adiponectin, epidemiology

## Abstract

A population-based case–control study of lymphomas in England collected height and weight details from 699 non-Hodgkin's lymphoma (NHL) cases and 914 controls. Obesity, defined as a body mass index (BMI) over 30 kg m^−2^ at five years before diagnosis,, was associated with an increased risk of NHL (OR=1.5, 95% CI 1.1–2.1). The excess was most pronounced for diffuse large B-cell lymphoma (OR=1.9, 95% CI 1.3–2.8). Genetic variants in the leptin (*LEP* 19G>A, *LEP* −2548G>A) and leptin receptor genes (*LEPR* 223Q>R), previously shown to modulate NHL risk, as well as a polymorphism in the energy regulatory gene adiponectin (*APM1* 276G>T), were investigated. Findings varied with leptin genotype, the risks being decreased with LEP 19AA (OR=0.7, 95% CI 0.5–1.0) and increased with LEP −2548GA (OR=1.3, 95% CI 1.0–1.7) and −2548AA (OR=1.4, 95% CI 1.0–1.9), particularly for follicular lymphoma. These genetic findings, which were independent of BMI, were stronger for men than women.

Apart from a small proportion caused by severe immunosuppression, the cause of the majority of non-Hodgkin's lymphomas (NHL) remains largely unknown. Both over- and undernutrition can suppress immunity via for example leptin, leptin receptor and adiponectin, which are expressed by adipocytes to regulate food intake and energy expenditure (Marti *et al*, 2001; Samartin and Chandra 2001). Accordingly, it has been suggested that anthropometric measures, reflecting the degree of adiposity, as well as polymorphisms in genes associated with energy homeostasis such as leptin (*LEP*), leptin receptor (*LEPR*) and adiponectin (*APM1*), may increase NHL risk. However, while positive associations have been reported between NHL and anthropometric characteristics (Holly *et al*, 1999; Calle *et al*, 2003; Bahl *et al*, 2004; Pan *et al*, 2004), others have not (Paffenbarger Jr *et al*, 1978; Whittemore *et al*, 1985; Franceschi *et al*, 1989; La Vecchia *et al*, 1990; Zhang *et al*, 1999; Cerhan *et al*, 2002; Chang *et al*, 2005). Furthermore, polymorphisms in the *LEP* (an A to G nucleotide (nt) change at position 19 in the 5′-untranslated region (Hager *et al*, 1998), and a G to A substitution at nt −2548 upstream of the ATG start site (Mammes *et al*, 1998)) and *LEPR* (an A to G transition at nt 668 from the start codon that converts glutamine to arginine at codon 223 (223Q>R) (Gotoda *et al*, 1997)) genes were recently shown to modulate NHL risk (Skibola *et al*, 2004).

Anthropometric and genetic findings from a large population-based case–control study of NHL carried out in England during the 1990s are presented here. Specifically, the potential aetiological role of body mass index (BMI) and the polymorphisms *LEP* 19A>G, *LEP* −2548G>A, and *LEPR* 223Q>R, as well as a polymorphism in the *APM1* gene (a G to T substitution at nt 276 downstream of the ATG start site denoted *APM1* 276G>T (Hara *et al*, 2002)), are investigated.

## MATERIALS AND METHODS

Details of this case–control study are described elsewhere (Willett *et al*, 2004). Briefly, cases were patients aged 18–64 years newly diagnosed with lymphoma between January 1998 and March 2001. Diagnoses were pathologically confirmed and coded to the World Health Organisation Classification (Fritz *et al*, 2000), and patients were ineligible if they had a previous diagnosis of lymphoma or HIV. One control per case, individually matched on sex and date of birth, was randomly selected from population registers. All participants were interviewed in person, and asked to provide a blood sample for research purposes. Study subjects were assigned an area-based indicator of deprivation by coding the enumeration districts (ED) where subjects resided at diagnosis/reference date to categories of the 1991 census-derived Townsend scores of EDs across England and Wales (Townsend *et al*, 1988). The study was conducted with the ethical approval of the United Kingdom Multi-Regional Ethical Committee.

At interview, participants were asked what their height and weight was at 5, 10 and 20 years prior to diagnosis/reference date. Body mass index was computed by dividing weight in kilograms by the square of height in metres. Height was categorised based on the observed distributions among controls who were aged 18 years or over at each time point. Body mass index was grouped into underweight (<18.5 kg m^−2^), normal (18.5–24.99 kg m^−2^), grade 1 overweight (25–29.99 kg m^−2^), and grades 2 and 3 overweight (30 kg m^−2^ or more) (WHO Expert Committee on Physical Status, 1995).

DNA was isolated from peripheral blood mononuclear cells using a modified phenol–chloroform extraction and was quantified using PicoGreen® dsDNA Quantitation kits (Molecular Probes, Eugene, OR, USA), according to the manufacturer's specifications. Samples, blinded to case–control status, were genotyped using Taqman®-based assays designed by Applied Biosystems (ABI) (Applied Biosystems, Foster City, CA, USA). Reactions were performed on an ABI 7700 or GeneAmp PCR 9700 System for 10 min at 95°C, then 40 cycles of 95°C for 15 s and 60°C for 1 min; a post-PCR plate read on the ABI 7700 system was used to determine genotype. Genotyping probes and primers used are listed in Table 1. To ensure reproducibility of the genotyping procedure, replicate quality control samples were included with an agreement rate of over 99%.

Interviews were conducted with 912 lymphoma patients and 919 controls, 75% of cases and 71% of the controls identified, and blood samples were received from 843 (94%) cases and 829 (95%) controls who were interviewed. Among the 820 Caucasian cases and 826 Caucasian controls who gave a blood sample, 736 (90%) cases and 754 (91%) controls had sufficient DNA to genotype all SNPs under investigation; insufficient or no DNA was available to test all four SNPs for 84 (10%) cases and 72 (9%) controls. The analysis presented here for height and BMI are restricted to 699 Caucasian cases with a confirmed diagnosis of NHL and 914 Caucasian controls, all aged 18–59 at 5 years prior to diagnosis/reference date, while the analysis of the genotyping data includes 593 Caucasian cases with a confirmed diagnosis of NHL and 754 Caucasian controls. All analyses include controls matched to NHL and Hodgkin's lymphoma (HL) cases.

Odds ratios (OR) and 95% confidence intervals (CI), adjusted for sex, age and region, were calculated using unconditional logistic regression; risk estimates for matched analyses were also computed using conditional logistic regression (Breslow and Day, 1980). Tests for trend and for interaction were conducted using the likelihood ratio test. Haplotype frequencies were estimated using log-linear modelling embedded within an expectation maximisation algorithm. All analyses were performed using Stata (StataCorp, 2003).

## RESULTS

Among interviewed cases with a confirmed diagnosis, 317 (45%) were diagnosed with diffuse large B-cell lymphoma (DLBCL), 228 (33%) with follicular lymphoma (FL), and 154 (22%) with rarer forms of NHL. A higher proportion of men than women were diagnosed with NHL and this increased with age. The majority of controls (76%) were individually matched to cases on sex and age, and although cases tended to be older due to the inclusion of controls matched to patients diagnosed with Hodgkin's lymphoma, there was little difference between the mean ages of cases (53.5 years) and controls (51.8 years). Cases and controls, who were interviewed or those who were genotyped for energy homeostasis SNPs, were similar with respect to deprivation (data not shown).

Table 2 presents the height distributions of cases and controls. Compared to those whose reported height was between 1.74 and 1.80 m, men of taller or shorter stature were not at increased risk of NHL, DLBCL or FL. With the exception of an increased risk for women less than 1.53 m tall (OR=2.2, 95% CI 0.9–5.1, *P*=0.07), no excess of NHL was observed for women who were over or under 1.58–1.68 m tall. This elevated risk in the smallest females was evident for DLBCL (OR=2.9, 95% CI 0.9–9.0, *P*=0.07) and not FL, where rather, risks were decreased among women of above average height (1.69–1.73 m: OR=0.5, 95% CI 0.3–1.0, *P*=0.06; >1.73 m: OR=0.4, 95% CI 0.2–1.0, *P*=0.06).

Using the WHO categorisation of BMI (Table 3), risks were estimated relative to cases and controls who were of normal weight-for-height. Persons who were underweight were at decreased risk (OR=0.5, 95% CI 0.2–1.5, *P*=0.22), while those who were grade 1 overweight (OR=1.2, 95% CI 1.0–1.5, *P*=0.10), or grades 2 or 3 overweight (OR=1.5, 95% CI 1.1–2.1, *P*=0.01) were at increased risk of NHL. Tests for trend were significant for NHL and DLBCL but not for FL; a 5 kg m^−2^ rise in BMI increasing the risk of DLBCL by 30% (95% CI 1.2–1.5, *P*<0.0001). These patterns were similar for men and women (data not shown). With respect to age at diagnosis, there was some suggestion that the risk of NHL associated with being markedly overweight may be more pronounced at younger ages: the OR falling from 2.7 (95% CI 1.2–5.8, *P*=0.01), to 1.8 (95% CI 1.0–3.3, *P*=0.04) to 1.2 (95% CI 0.8–1.9, *P*=0.42) in those aged under 45, 45–54 and 55 years or more, respectively. Further, among the under 45 s, the finding was stronger for DLBCL (OR=3.0, 95% CI 1.2–7.5, *P*=0.02) than for FL (OR=1.8, 95% CI 0.5–6.3, *P*=0.33). Within the DLBCL subtype, risks were greater among men aged less than 45 (OR=4.7, 95% CI 1.1–19.4, *P*=0.03 based on five cases and four controls) than among women of the same age group (OR=2.5, 95% CI 0.7–8.6, *P*=0.15 based on five cases and 10 controls). Analyses were repeated using anthropometric data at 10 and 20 years before diagnosis/reference date, and using the individually matched controls alone and conditional logistic regression, and the findings were similar (data not shown).

Genotype distributions for cases and controls for the *LEP* 19G>A, *LEP* −2548G>A, *LEPR* 223Q>R and APM1 276G>T polymorphisms are shown in Table 4. Control genotype distributions for all SNPs were in Hardy–Weinberg equilibrium. Relative to LEP 19GG, the LEP 19AA genotype was inversely associated with FL (OR=0.5, 95% CI 0.3–0.8, *P*=0.01), but not DLBCL (OR=0.9, 95% CI 0.6–1.4, *P*=0.70). For LEP −2548G>A, an increased risk for NHL was observed among carriers of the LEP −2548GA (OR=1.3, 95% CI 1.0–1.7, *P*=0.03) and *LEP* −2548AA genotypes (OR=1.4, 95% CI 1.0–1.9, *P*=0.04), particularly for FL (*LEP* −2548GA: OR=1.6, 95% CI 1.1–2.3, *P*=0.01; *LEP* −2548AA: OR=1.4, 95% CI 0.9–2.3, *P*=0.12) when compared to LEP −2548GG carriers. Stratifying by sex revealed that among men, the *LEP* 19AA genotype was associated with a reduced risk of NHL (OR=0.6, 95% CI 0.4–1.0, *P*=0.06) and FL (OR=0.3, 95% CI 0.1–0.7, *P*=0.005), while the *LEP* −2548AA genotype was associated with an increased risk of NHL (OR=1.6, 95% CI 1.1–2.5, *P*=0.02), DLBCL (OR=1.6, 95% CI 1.0–2.7, *P*=0.07) and FL (OR=1.8, 95% CI 0.9–3.5, *P*=0.10). No associations were found in women with the exception of an increased risk of FL among carriers of the *LEP* 223RR (OR=1.9, 95% CI 1.0–3.6, *P*=0.04) relative to the *LEP* 223QQ genotype. There were no differences in genotype distributions between cases and controls for the *APM1* 276G>T SNP. Tests for interactions between SNPs were not statistically significant. LEP 19G>A and *LEP* −2548G>A among controls, with estimated haplotype frequencies of −2548G/19A, 41.5%; −2548G/19G, 18.5%; −2548A/19G, 39.2%; and −2548A/19A, 0.8%, were in linkage disequilibrium (*D*=0.95), but despite the individual SNP effects, no associations between haplotypes and either NHL as a whole or FL in particular emerged.

Risks associated with BMI among interviewed and genotyped subjects were similar, with the OR of NHL among the 593 cases and 754 controls who were genotyped being 1.2 (95% CI 0.9–1.5, *P*=0.16) and 1.4 (95% CI 1.0–2.0, *P*=0.08) for being grade 1 and grades 2 or 3 overweight, respectively. To assess whether the risk associated with the energy homeostasis polymorphisms differed by BMI, analyses were repeated stratifying the genotyping data by WHO categories of BMI. The risks associated with the variant genotypes did not increase with rising grades of obesity; for example, the risk of NHL did not incline among persons carrying the LEP −2548AA genotype with increasing weight-for-height, from normal weight (OR=1.3, 95% CI 0.9–2.0, *P*=0.19), through grade 1 overweight (OR=1.5, 95% CI 0.9–2.5, *P*=0.09), to grades 2 and 3 overweight (OR=0.9, 95% CI 0.3–2.5, *P*=0.88). Similarly, there was no suggestion that the risk estimates associated with the leptin SNPs varied between persons of normal BMI, grade 1, or grades 2 and 3 obesity (data not shown).

## DISCUSSION

Here we present evidence that obesity and variants in the *LEP* gene may be important in the pathogenesis of NHL. Specifically, we found an association between NHL and excess adiposity estimated using BMI 5 years prior to diagnosis in both men and women, with a greater risk found among patients diagnosed at younger ages (<45 years). Risks were elevated for the two most common NHL subtypes, but the association remained statistically significant only for DLBCL. In contrast, the *LEP* 19G>A and *LEP* −2548G>A polymorphisms altered the risk of FL, particularly among men. These SNPs were not associated with risk of DLBCL and, generally, no associations were observed for *LEPR* 223Q>R and APM1 276G>T. Given the increasing obesity and lymphoma rates worldwide, our findings may have important public health implications.

While several studies have suggested that the risk of NHL associated with BMI varies little with age and sex (Holly *et al*, 1999; Calle *et al*, 2003; Pan *et al*, 2004; Chang *et al*, 2005), our study is the first to examine risk by disease subtype, sex and age. With respect to the former, we, like others (Cerhan *et al*, 2002; Skibola *et al*, 2004; Chang *et al*, 2005), found that the risk of DLBCL rose with increasing BMI, but patterns for FL were less consistent. Non-Hodgkin's lymphoma, and particularly DLBCL, is more common in men than women and the incidence increases with age above 25 years (Clarke and Glaser, 2002; Cartwright *et al*, 2005). Our observation for DLBCL in young men is based on small numbers, but if real, could reflect the effects of weight gain early in adulthood. Not only does body fat, and hence BMI, increase with age, but the site of fat deposition may also be important – men being most likely to accumulate abdominal fat than women (WHO Consultation on Obesity, 2000). Better markers of central, rather than total, adiposity would be waist circumference or waist-to-hip ratio (WHO Consultation on Obesity, 2000), which, up to now, have only been reported in one study of women, but no association with NHL was found (Cerhan *et al*, 2002). If weight gain in early adulthood is not responsible, our results could instead suggest that obesity from childhood is a risk factor for NHL. It seems unlikely however that nutrition during childhood, linked to adult stature (Silventoinen, 2003), increases NHL risk, since generally little evidence of an association with height has been presented either here or elsewhere (Whittemore *et al*, 1985; La Vecchia *et al*, 1990; Zhang *et al*, 1999; Cerhan *et al*, 2002). Alternatively, it may be that an underlying genetic component of obesity is involved in the pathogenesis of NHL.

Leptin, an adipokine which circulates at levels proportional to adipose tissue mass, regulates immune function as well as nutritional status (Otero *et al*, 2005). As circulating levels increase, leptin acts to modulate food intake, but in obesity, its rise with increasing body fat has limited effect on satiety, suggesting negative regulators of leptin and insulin signalling, such as leptin receptor and adiponectin, are present (Bell *et al*, 2005). In the absence of measured plasma levels before the diagnosis of NHL, long-term variation in production of these adiponectins may be indicated by the polymorphisms investigated here, since the *LEP* 19G, *LEP* −2548A and *LEPR* 223R alleles have been associated with elevated leptin levels, and the *APM1* 276T allele with lower adiponectin levels (Hoffstedt *et al*, 2002; van Rossum *et al*, 2003; Filippi *et al*, 2004). Further, these polymorphisms have been linked with an obese phenotype in some Caucasian populations (Li *et al*, 1999; Mammes *et al*, 2000; Quinton *et al*, 2001; Yiannakouris *et al*, 2001; Nieters *et al*, 2002; Filippi *et al*, 2004; Jiang *et al*, 2004). Previously, Skibola *et al* (2004) reported that, relative to the *LEP* 19AA genotype, carriers of the *LEP* 19G allele were at increased risk of NHL, particularly FL (OR=1.9, 95% CI 1.0–3.6). Using *LEP* 19GG as the referent group, the recalculated FL risk estimate for the *LEP* 19AA genotype is 0.6 (95% CI 0.3–1.3), similar to the OR of 0.5 (95% CI 0.3–0.8) presented here. As has been observed elsewhere, the BMIs of our controls were not correlated with *LEP* 19G>A (*ρ*=0.014, *P*=0.70) (Karvonen *et al*, 1998; Lucantoni *et al*, 2000), *LEP* −2548G>A (*ρ*=−0.013, *P*=0.72) (Mammes *et al*, 1998; Le Stunff *et al*, 2000), *LEPR* 223Q>R (*ρ*=−0.008, *P*=0.83) (Gotoda *et al*, 1997; Rosmond *et al*, 2000; Wauters *et al*, 2001) or *APM1* 276G>T (*ρ*=−0.024, *P*=0.51) (Menzaghi *et al*, 2002; Fumeron *et al*, 2004). Moreover, this and the previous report by Skibola *et al* found little evidence that risks associated with BMI varied by genotype.

Obesity can induce a state of leptin and insulin resistance, chronic low-grade inflammation, and increased leptin, tumour necrosis factor (TNF)-α, IL-6 and C reactive protein serum levels (Marti *et al*, 2001). These proinflammatory mediators can activate a number of signalling pathways including nuclear factor (NF)-*κ*B that result in antiapoptotic and proliferative behaviour in B-cells. Recently, DLBCL subtypes have been characterised by NF-*κ*B gene expression signatures, suggesting the significance of activation of this transcription factor in DLBCL pathogenesis (Rosenwald and Staudt, 2003). Increased risk of FL associated with a high leptin producer phenotype (*LEP*-2548AA), or reduced risk associated with a low leptin producer phenotype (*LEP* 19AA), may relate to leptin's action in upregulating expression of the antiapoptotic *BCL*-*2* protein in B-cells through the Janus kinase (JAK)/signal transducer and activator of transcription (STAT) pathways. Most FL have a t(14;18) chromosome translocation resulting in *BCL-2* dysregulation and overexpression. Leptin also increases expression of matrix metalloproteinases and tissue inhibitors of metalloproteinases, which are associated with aggressive disease, neoplastic growth and angiogenesis in B-cell lymphomas.

This study has a high level of case ascertainment and diagnostic confirmation. As with all case–control studies of this type, however, the possibility that the findings may reflect bias in the case and/or control populations needs to be considered. In our study, participants who resided in affluent areas were, on average, of smaller stature and greater BMI than those who resided in deprived areas. This pattern agrees with that seen in most national surveys (Joint Health Surveys Unit, 2003), and it is possible that the increased risks may have arisen from differential case–control participation. However, adjustment for deprivation did not alter the risk estimates for height or BMI. A further limitation relates to the self-reported nature of the anthropometric data analysed. Compared to a sample of the British population with anthropometric measurements (National Center for Social Research, 2004), our controls were taller and lighter, leading to a reduction in their derived BMI. It is, however, unlikely that cases estimated their height and weight differently from controls, as the hypothesis that obesity is related to NHL is not widely known.

In conclusion, our findings raise the possibility that obesity may increase risk of NHL as a whole, and DLBCL risk in particular. Our findings were most marked among men and those diagnosed at a comparatively young age (<45 years). Although the SNPs involved in energy homeostasis investigated in our study did not modify the risk of NHL associated with obesity, independent effects were seen for FL with the *LEP* 19G>A and *LEP* −2548G>A SNPs, irrespective of adiposity. Previous reports of NHL and BMI have been inconsistent, with little investigation of disease subtypes. New initiatives, aimed at pooling data from studies around the world including the one reported here (Boffetta *et al*, 2003), will hopefully provide further insight into the association between lymphoma and BMI.

## Figures and Tables

**Table 1:** Probes and primers for *LEP* 19G>A, *LEP* −2548G>A, *LEPR* 223Q>R, and *APM1* 276G>T polymorphisms

**SNP**	**dbSNP rs number**	**Probe/primer**	**5′-3′ sequence**
*LEP* 19G>A	rs2167270	F	GGAGCCCCGTAGGAATCG
		R	CCAGCAGAGAAGGAGGAAGGA
		A	VIC-AACCGTTGGCGCTG
		G	6FAM-AACCGCTGGCGCT
			
*LEP* −2548G>A	rs7799039	F	TCCCGTGAGAACTATTCTTCTTTTG
		R	CCTGCAACATCTCAGCACTTAGG
		G	VIC-AGGATCAGCGCAAC
		A	6FAM-ATCAGTGCAACCCT
			
*LEPR* 223Q>R	rs1137101	F	GTTTGAAAATCACATCTGGTGGAGTA
		R	CATATTTATGGGCTGAACTGACATTAG
		Q	VIC-AGGTGACTGGAAAAT
		R	6FAM-AGGTGACCGGAAAA
			
*APM1* 276G>T	rs1501299	F	TTTCATCACAGACCTCCTACACTGA
		R	TCTCCCTGTGTCTAGGCCTTAGTTA
		GG	VIC-TGAATGCCTTCATATAGT
		T	FAM-ATGAATGACTTCATATAGTT

**Table 2:** Number of cases and controls, adjusted odds ratios and 95% confidence intervals for height

		**NHL^b^**			**DLBCL^b^**			**FL^b^**		
**Height^a^**	**Control**	**Case**	**OR^c^**	**95% CI**	**Case**	**OR^c^**	**95% CI**	**Case**	**OR^c^**	**95% CI**
*Males*	*495*	*361*			*167*			*103*		
⩽1.65	26	19	1.0	0.5–1.9	11	1.4	0.7–3.0	4	0.7	0.2–2.1
1.66–1.73	139	103	1.0	0.7–1.4	45	1.1	0.7–1.7	32	1.0	0.6–1.7
**1.74**–**1.80**	**214**	**151**	**1**	—	**63**	**1**	—	**47**	**1**	—
1.81–1.88	104	82	1.1	0.8–1.6	45	1.5	0.9–2.3	19	0.8	0.5–1.5
>1.88	12	6	0.7	0.3–1.9	3	0.8	0.2–3.1	1	0.4	0.0–3.0
										
*Females*	*419*	*338*			*150*			*125*		
⩽1.52	43	35	2.2	0.9–5.1	10	2.9	0.9–9.0	19	1.1	0.4–3.5
1.53–1.57	82	74	1.1	0.6–1.9	28	1.5	0.6–3.3	34	0.9	0.5–1.8
**1.58**–**1.68**	**226**	**171**	**1**	—	**86**	**1**	—	**55**	**1**	—
1.69–1.73	56	38	1.0	0.6–1.6	18	1.6	0.8–3.4	11	0.5	0.3–1.0
>1.73	12	20	0.9	0.5–1.6	8	1.4	0.6–3.4	6	0.4	0.2–1.0

a Height, in metres, at diagnosis/reference date.

b NHL=non-Hodgkin's lymphoma; DLBCL=diffuse large B-cell lymphoma; FL=follicular lymphoma.

c Odds ratios adjusted for age and region estimated using unconditional logistic regression.

**Table 3:** Number of cases and controls, adjusted odds ratios and 95% confidence intervals by age for body mass index

		**NHL^b^**			**DLBCL^b^**			**FL^b^**		
**BMI^a^**	**Control**	**Case**	**OR^c^**	**95% CI**	**Case**	**OR^c^**	**95% CI**	**Case**	**OR^c^**	**95% CI**
*Total*	*914*	*699*			*317*			*228*		
Underweight	14	5	0.5	0.2–1.5	2	0.5	0.1–2.0	0	0	
**Normal**	**510**	**347**	**1**	*—*	**159**	**1**	—	**120**	**1**	*—*
Grade 1	298	247	1.2	1.0–1.5	99	1.1	0.8–1.4	80	1.2	0.9–1.6
Grades 2 and 3	89	96	1.5	1.1–2.1	54	1.9	1.3–2.8	27	1.2	0.8–2.0
										
<*45 years*	*202*	*107*			*60*			*29*		
Underweight	4	1	0.5	0.1–5.0	1	1.1	0.1–11.0	0		
**Normal**	**133**	**60**	**1**	—	**32**	**1**	—	**18**	**1**	—
Grade 1	51	26	1.2	0.6–2.1	15	1.2	0.6–2.4	6	1.1	0.4–3.1
Grades 2 and 3	14	17	2.7	1.2–5.8	10	3.0	1.2–7.5	4	1.8	0.5–6.3
										
*45 to* <*55 years*	*297*	*243*			*107*			*93*		
Underweight	8	1	0.2	0.0–1.3	0			0		
**Normal**	**174**	**127**	**1**	—	**58**	**1**	—	**49**	**1**	—
Grade 1	91	83	1.4	0.9–2.0	32	1.1	0.7–1.9	31	1.3	0.7–2.1
Grades 2 and 3	24	31	1.8	1.0–3.3	16	2.0	1.0–4.1	13	1.9	0.9–4.0
										
*55 to* <*65 years*	*415*	*349*			*150*			*106*		
Underweight	2	3	1.9	0.3–11.3	1	1.5	0.1–16.6	0		
**Normal**	**203**	**160**	**1**	—	**69**	**1**	—	**53**	**1**	—
Grade 1	156	138	1.1	0.8–1.6	52	1.0	0.6–1.5	43	1.2	0.7–1.8
Grades 2 and 3	51	48	1.2	0.8–1.9	28	1.6	1.0–2.8	10	0.8	0.4–1.6

a Body mass index (BMI) at 5 years prior to diagnosis/reference date where BMI categorised as underweight: <18.5 kg m^−2^; normal: 18.5–24.99 kg m^−2^; grade 1 overweight: 25–29.99 kg m^-2^; grades 2 and 3 overweight: ⩾30 kg m^−2^ (WHO Expert Committee on Physical Status, 1995). BMI was missing for four cases and three controls as weight was not reported.

b NHL=non-Hodgkin's lymphoma; DLBCL=diffuse large B-cell lymphoma; FL=follicular lymphoma.

c Odds ratios adjusted for age, sex and region estimated using unconditional logistic regression.

**Table 4:** Number of cases and controls, adjusted odds ratios, and 95% confidence intervals for energy homeostasis polymorphisms

		**NHL^a^**			**DLBCL^a^**			**FL^a^**		
**SNP^b^**	**Control**	**Case**	**OR^c^**	**95% CI**	**Case**	**OR^c^**	**95% CI**	**Case**	**OR^c^**	**95% CI**
*Total:*	*754*	*593*			*270*			*210*		
*LEP 19G*>*A*										
**GG**	**275**	**235**	**1**	—	**104**	**1**	—	**86**	**1**	—
AG	357	276	0.9	0.7–1.1	123	0.9	0.7–1.2	102	0.9	0.6–1.3
AA	122	79	0.7	0.5–1.0	43	0.9	0.6–1.4	20	0.5	0.3–0.8
										
*LEP -2548G*>*A*										
**GG**	**260**	**170**	**1**	—	**81**	**1**	—	**55**	**1**	—
GA	348	294	1.3	1.0–1.7	128	1.2	0.9–1.6	113	1.6	1.1–2.3
AA	145	127	1.4	1.0–1.9	59	1.3	0.9–1.9	42	1.4	0.9–2.3
										
*LEPR 223Q*>*R*										
**QQ**	**234**	**188**	**1**	—	**88**	**1**	—	**60**	**1**	—
QR	387	306	1.0	0.8–1.2	144	1.0	0.7–1.3	104	1.0	0.7–1.5
RR	133	99	0.9	0.7–1.3	38	0.8	0.5–1.2	46	1.3	0.8–2.0
										
*APM1 276G*>*T*										
**GG**	**398**	**341**	**1**	—	**158**	**1**	—	**114**	**1**	—
GT	311	216	0.8	0.7–1.0	96	0.8	0.6–1.1	82	1.0	0.7–1.3
TT	45	35	0.9	0.6–1.5	16	0.9	0.5–1.6	13	1.0	0.5–2.0

a NHL=non-Hodgkin's lymphoma; DLBCL=diffuse large B-cell lymphoma; FL=follicular lymphoma.

b Test for Hardy–Weinberg equilibrium among controls: LEP 19G>A *χ*^2^=0.12, *P*=0.73; LEP −2548G>A *χ*^2^=2.17, *P*=0.14; LEPR 223Q>R *χ*^2^=1.55, *P*=0.21; APM1 276G>T *χ*^2^=2.41, *P*=0.12. Samples were not amplifiable for three cases when testing for LEP 19G>A; two cases and one control for LEP −2548G>A; and one case for APM1 276G>T.

c Odds ratios adjusted for sex, age and region estimated using unconditional logistic regression.
